# The impact of poly-traumatization on treatment outcomes in young people with substance use disorders

**DOI:** 10.1186/s12888-021-03129-x

**Published:** 2021-03-08

**Authors:** Sidsel Karsberg, Morten Hesse, Michael Mulbjerg Pedersen, Ruby Charak, Mads Uffe Pedersen

**Affiliations:** 1 Centre for Alcohol and Drug Research, Aarhus University, Bartholins Allé 10, 8000 Aarhus, Denmark; 2 Department of Psychological Science, The University of Texas Rio Grande Valley, 1201 W. University Dr, Edinburg, TX USA

**Keywords:** Substance abuse, Treatment outcome, Trauma, Victimization, Cannabis

## Abstract

**Background:**

It is believed that clients with psychological trauma experiences have a poor prognosis with regard to treatment participation and outcomes for substance use disorders. However, knowledge on the effect of the number of trauma experiences is scarce.

**Methods:**

Using data from drug use disorder (DUD) treatment in Denmark, we assessed the impact of having experienced multiple potentially traumatic experiences on DUD treatment efficacy. Baseline and follow-up data from 775 young participants (mean age = 20.2 years, standard deviation = 2.6) recruited at nine treatment centers were included in analyses.

**Results:**

Analyses showed that participants who were exposed multiple trauma experiences also reported a significantly higher intake of cannabis at treatment entry, and a lower well-being score than participants who reported less types or no types of victimization experiences. During treatment, patients with multiple types of trauma experiences showed a slower rate of reduction of cannabis than patients with few or no trauma experiences. The number of trauma types was not associated with number of sessions attended or the development of well-being in treatment.

**Conclusion:**

Overall, the results show that although traumatized youth in DUD treatment show up for treatment, helping them to reduce substance use during treatment is uniquely challenging.

**Trial registration:**

ISRCTN88025085, date of registration: 29.08.2016, retrospectively registered.

## Background

Trauma experiences refers to serious adverse events that can instantaneously cause severe distress, and may lead to the syndrome known as post-traumatic stress disorder (PTSD). Trauma experiences include life-threatening situations, such as serious accidents, and also interpersonal trauma such as serious neglect, sexual abuse, or prolonged abuse or bullying. General population surveys conducted in 24 countries indicate that over 70% of individuals report a traumatic event during their lifetime [1]. However, experience of a traumatic event does not always lead to PTSD, but this near ubiquitous exposure can adversely affect psychological functioning and behavior [2, 3].

### Traumatization and poly-traumatization

Recently, researchers and clinicians have increasingly begun to focus on a particularly high-risk sub-group of children and youth who has been exposed to several types of trauma, rather than just a single type of traumatic event [4–6]. The theoretical foundation of this new focus is the cumulative risk theory which states that the higher the number of risk factors a person is exposed to, the higher the potential for a negative outcome [7] and the underlying dose response model which illustrate that the accumulation of traumatic events is often a stronger predictor of outcomes than single event exposure [8].

Individuals with a history of poly-traumatization have been shown to continue to experience additional victimization [9], severe and persistent biopsychosocial impairment [10, 11], and are at increased risk of developing negative behavioral patterns such as delinquency and substance abuse compared to individuals with few or no trauma experiences [12, 13]. In a study of youth involved in the juvenile justice system, investigators conducted a latent class analysis derived from 19 types of adversity, including different forms of violence exposure produced three unique classes. Among the three classes that emerged, the poly-trauma group was distinct from other adversity groups in the presence of severe emotional and behavioral problems [14]. In another recent longitudinal study that included 1186 adolescents, poly-victimization was linked to lower levels of social support, self-esteem, and mastery [15]. However, in recent studies that examine the accumulative effect of trauma victimization, certain specific types of traumatic exposures, for instance, child sexual abuse have also been shown to account for impairment over and above the cumulative impact of the number of specific exposure types [16–18]. None-the-less, the established links between multiple types of trauma, negative behavioral patterns, and cognitive functions may carry important implications for substance abuse treatment, particularly in relation to treatment efficacy.

### Traumatization and substance use treatment

The link between trauma experiences and substance use disorders (SUD) is well established [4]. In surveys of adolescents receiving treatment for substance abuse, between 60% and 87% of patients reported a history of exposure to trauma [5, 6]. Moreover, studies show that 37% to 52% of SUD patients have a lifetime diagnosis of PTSD [7, 8]. In particular, the literature has focused on experiences of childhood abuse as an underlying risk factor for substance use behaviors [9–11]. Examining the effect of trauma history on SUD treatment outcomes is important, because the treatment of SUD patients with a trauma history may need to be adjusted to the needs of the patient based on their trauma experiences [19, 20]. For instance, treatment may lead to exposure to cues related to the traumatic events. This may, if unaddressed, produce symptoms of anxiety or anger, in turn leading to patient attrition, or reinitiating substance use to cope with intruding memories and avoidance symptoms [21, 22].

However, overall, the influence of previous trauma experiences on the effect of SUD treatment is unclear. A number of studies have indeed indicated that individuals with past trauma experiences, have poorer outcomes of substance abuse treatment including worse treatment adherence and less improvement during treatment [23–27]. Yet, other studies have found that past trauma experiences are not a predictor of poor prognosis or missing appointments in SUD treatment [28–30].

The majority of studies that have examined associations between specific trauma experiences and substance use disorder (SUD) treatment outcomes, have focused on single types of childhood trauma such as physical or sexual abuse [30–33]. Childhood trauma as an overall category has been found to be a predictor for earlier drop-out from SUD treatment [34], higher rates of relapse [35] and less improvement in treatment measured by drug use and psychological functioning [36]. Also, Parolin and colleagues found that young drug users receiving drug use disorder (DUD) treatment who had been exposed to parental substance abuse during their developmental age, had elevated rates of neuropsychological impairments, particularly affecting the attentive and executive functions, compared to drug users who were not exposed to parental substance abuse [37]. However, to our knowledge, existing studies have not controlled for the effect of other trauma exposure on treatment outcomes and therefore it is unclear whether it is indeed the specific type of trauma or an accumulative effect of trauma exposure that is associated with treatment effect.

In light of the inconsistent findings, there is a need for additional research that examines the role of past trauma experiences in substance abuse treatment settings. In particular, very few studies have examined the influence of the number of trauma experiences in relation to outcomes of DUD treatment. To strengthen knowledge in the substance abuse treatment field, this study aimed to examine the effect of poly-traumatization on treatment effect in a Danish treatment for DUD based on cognitive-behavioral therapy.

Since the word trauma holds multiple meanings, it is important to point out that the events referred to as trauma in the present study are only potentially traumatic, meaning that they may or may not have caused trauma symptoms. The same applies for the term poly-traumatization, which in this study covers multiple types of trauma experiences and not the level of trauma symptomatology. Recent trauma studies have used the terms correspondingly [38, 39].

### Study aims

Based on the cumulative risk theory and existing studies that link accumulative trauma to a lower SUD treatment effect, as well as various negative psychosocial and behavioral factors that are relevant for treatment outcome, such as self-esteem, social support and mastery [15], attentive and executive functions [30], and severe emotional and behavioral problems [5, 12], the overall research question for the present paper was whether participants with trauma exposure, and in particular a high number of traumatic experiences, would be less likely to benefit from DUD treatment than participants with no or few trauma experiences. This focus led to the following three hypotheses:

1. Trauma history would be associated with a slower reduction in cannabis use during treatment in an approximately dose-dependent fashion, in that the fastest decline in cannabis use would be seen among participants with no traumatic history, and the slowest among those with poly-traumatization.
2. Trauma history would be associated with a slower improvement in well-being during treatment in a similar dose-dependent fashion.
3. Treatment attendance would be associated with trauma history, in that patients with zero trauma experiences would attend the most sessions, followed by patients with few types of trauma, and that the patients with the highest number of trauma experiences would attend the fewest sessions.

Since similar studies have shown that factors such as gender [40, 41], socio economic status [42], immigrant status/ethnic minority status [43], and age [44, 45] may play a great role in relation to SUD treatment outcome, we controlled for the effect of these factors in the present study.

## Methods

### Setting and participants

The study combined data from two sources: one was a randomized controlled clinical trial [46] that examined the effect of a motivational enhancement and CBT-based intervention. Participants were recruited by staff at nine participating treatment centers. The second source was data collected from the same nine sites during the post-trial phase of the clinical trial, when treatment components were implemented in day-to-day practice^1^.

All participating sites offered outpatient treatment for youth with illicit DUDs in agreement with the Danish Consolidation Act on Social Services under Part 18, § 101. According to the act, treatment must be offered without co-payment, and within two weeks after clients have contacted the treatment services.

Participants were eligible if they were between the ages of 15 and 25 years, and had contacted the treatment center with the intention of entering treatment for DUD. Clients were excluded if they sought treatment for an opioid use disorder, had known active psychotic disorders, known cognitive deficits, and/or were otherwise deemed not safe to participate (e.g., had recently engaged in violent actions against staff). The reason for excluding participants with opoid use disorders was that treatment for this particular group is often focused on substitution medicine and this focus is very important throughout treatment. It was therefore assessed that the offered treatment would cover this essential focus for this particular group well enough. The demographic characteristics are summarized in Table 1.

**Table 1:** Baseline characteristics total sample

	n = 775
Female gender	22.7% (*n* = 176)
Mean age	20.3 (2.65)
Immigrant	9.9% (n = 77)
Not in education, employment or training	36.5% (*n* = 283)
Days of cannabis use within last month	17.6 (11,34)
Binge drinking/typical week	0.82 (1.20)
Days of cocaine use within last month	1.15 (3.2)
Days of ecstasy/MDMA use within last month	0.42 (1.9)
Days of illegal behavior for profit within last month	2.44 (6.37)
Self-reported psychiatric diagnosis	34.7% (269)
Bullying	41.3% (*n* = 302)
Neglect	58.7% (*n* = 451)
Sexual assault	15.9% (*n* = 124)
Physical assault	52.1% (*n* = 414)
Threats	63.3% (*n* = 482)
Accident	31.5% (*n* = 243)
Severe disease	33.4% (*n* = 259)
Death of parent or sibling	14.7% (*n* = 104)
0 potentially traumatic events	8.3% (*n* = 64)
1–4 potentially traumatic events	69.7% (n = 540)
5–8 potentially traumatic events	22.1% (n = 171)

### Procedures

Eligible clients were invited to take part in the clinical trial by clinical staff at each of the nine treatment centers.

From September 2014 to May 2016, clients were invited to participate in the clinical trial, and from the end of May 2016 and until October 2017, all eligible clients were asked if they would consent to the use of their assessment data for research purposes and quality assurance.

### Interventions

All participating clients were offered 12 sessions of manual-based counselling incorporating a combination of cognitive behavioral therapy and motivational interviewing (see Table 2).

**Table 2:** Treatments offered in the trial and implementation phases

Trial Phase (2014–2016)				Implementation Phase (2016–2018)
*n* = 114	*n* = 113	*n* = 112	*n* = 121	*n* = 315
12 sessions standard treatment (MI/CBT)	12 sessions standard treatment (MI/CBT) + vouchers	12 sessions standard treatment (MI/CBT) + reminders	12 sessions standard treatment (MI/CBT) + vouchers + reminders	12 sessions standard treatment (MI/CBT) + reminders + vouchers (optional)

In addition to this manual based counselling (standard treatment), some participants received vouchers for attendance, reminders of sessions, or both vouchers and reminders [46]. Based on the variety of treatment components, the potential effect of different treatment tracks was controlled for in our analyses (i.e., the four randomized groups and the post-trial phase).

### Measures

At the first counselling session after trial participation, all clients were assessed using the YouthMap assessment form [47]. The YouthMap assessment form is a comprehensive assessment tool, tailored for young people in treatment for psychoactive substance use disorders. The YouthMap covers background information, current substance use, past and current behavioral and mental problems, victimization experiences, peer and family relations amongst others. Additionally, during the treatment course all participants were assessed in each session with the TEM screening tool [48, 49]. TEM is a routine outcome monitoring tool that consists of eight questions based on the feedback informed treatment approach [50, 51]; five questions related to drug use and three questions related to well-being. All responses were entered into a secure web-based interface at baseline by the involved researchers.

#### Lifetime exposure to traumatic events

To measure the type and number of past trauma experiences the following eight items from the YouthMap survey were selected: 1) Have you ever been the victim of bullying?”, 2) “Have you ever been the victim of neglect?”, 3) “Have you ever been the victim of a sexual assault?”, 4) “Have you ever been the victim of a physical assault?”, 5) “Have you ever received threats to your health or life?”, 6) “Have you ever been involved in a serious accident, such as a traffic accident or a work-related accident?”, 7) “Have you ever experienced that you, your parents, or your siblings were seriously ill.”, 8) “Have you ever experienced the death of a sibling or parent?” For bullying, the responses were rated on a five-point Likert scale from 1 (“Not at all”) to 5 (“extensively”), and all other trauma victimization items were dichotomized (yes/no).

Moreover, clinicians registered all completed counselling sessions and all non-attendances for their clients.

#### Outcomes

Three central outcomes of interest were considered for this study: 1) cannabis use during treatment, 2) psychological well-being during treatment, and 3) session attendance. Due to the small proportion of participants who used other types of drugs but not cannabis, we only included participants who reported cannabis use in the analyses of effects on drug intake.

Cannabis use during treatment was measured by asking about the number of times per day within the last week that the participant had smoked cannabis. Cannabis use was recoded into number of days with any cannabis use. Well-being was measured by a composite score using three questions: 1) “How well have you generally been feeling during the seven days?”, 2) “How well have you generally felt about your close relationships, e.g., family or close friends, during the past seven days?”, and 3) “How well have you generally felt socially, e.g. at work or school, during the past seven days?”. The three items are from the outcome rating scale (ORS) which consists of four items [52]. Previous studies have found that the ORS has high test-retest reliability, strong internal consistency, and moderate to good concurrent validity [52–54].

All three items were measured on a 10-point scale ranging from 0) “very bad” to 10) “very good”. To measure attendance each session up to session 12 was dummy-coded as attended or missed. Thus, it was possible for participants to have a pattern of attendance consisting of a mix of sessions attended (e.g., two attended, one missed, followed by nine attended).

#### Control variables

For the three regression variables, random intercepts were estimated for site and client. In addition, the analyses were controlled for gender, age (measured as an interval scale), not being in education, employment or training (single, dummy coded variable), and migration status (dummy coded as having at least one Danish parent versus all others).

### Analytic plan

To assess poly-traumatization, the sample was divided into three trauma categories based on the number of reported trauma experiences. The trauma types do not represent a scale, as each is a potentially independent event from the others, rather than different manifestations of the same underlying construct. For this reason, we chose to analyze the number of different traumatic events as an index [55]. That is, a measure of factors that may all individually contribute to the same phenomenon (in this case, being burdened by the impact of traumatic events). We used the eight items measuring trauma victimization. For seven of the eight trauma variables the response “yes” was coded as trauma victimization. For bullying, answers were only coded as trauma victimization if participants had responded “to some extent”, “a lot”, or “extensively”. Individuals were grouped as zero trauma experiences, one to four trauma experiences, or five or more trauma experiences.

A mixed effects Tobit regression was conducted for days of cannabis use. Days of cannabis use was censored at zero days (i.e., past week abstinence) and 7 days (i.e., daily use). The variables of interest were status in terms of trauma exposure measured on a nominal scale (i.e., zero trauma types, one to four trauma types, or five or more trauma types), and the interaction between trauma status and session. The interaction between trauma and session was included in order to assess the slope of days of cannabis use as a function of trauma categories (i.e. the number of different types of victimization).

A mixed effects logistic regression was conducted for session attendance. The variables of interest were again status in terms of trauma measured on a nominal scale (i.e., zero trauma types, one to four trauma types, or five or more trauma types), and the interaction between trauma status and session.

A linear mixed effects regression was conducted for well-being. The variables of interest were again status in terms of trauma measured on a nominal scale (i.e., zero trauma types, one to four trauma types, or five or more trauma types), and the interaction between trauma status and session.

Finally, two graphs were produced to illustrate the course of cannabis use and well-being throughout treatment. For use of cannabis and well-being, a fractional polynomial fit was used to assess days of cannabis use as a function of session, stratified by degree of poly-traumatization.

Missing data can influence the results of a prospective clinical study, when data is not missing completely at random. While there is no one solution to this issue, one way to reduce the influence of missing data is to conduct sensitivity analyses adjusting for factors that are likely to influence missingness [56]. While we cannot guarantee that we can identify all factors that are associated with missing data, we do know that randomization to any of the active treatment groups in our study increased attendance and reduced no-shows [57].

As a sensitivity analyses, all analyses were conducted controlling for treatment group (four randomized groups and post-trial status). Further sensitivity analyses were conducted controlling for the externalizing and internalizing scales of the YouthMap measure [58].

## Results

### Participants

A total of 775 participants were included in the study, of which 599 were male and 176 were female. The mean age was 20.2 years (standard deviation [SD] = 2.6, range = 15 to 25). At baseline, the participants reported smoking cannabis on average 17.6 days in the past 30 (SD = 11.3, range 0 to 30) and 4.04 days in the past 7 days (SD = 2.77, range 0–7). A total of 38% reported use of drugs or substances other than cannabis or alcohol.

### Poly-traumatization

Of the participants, 64 (8.3%) had never experienced any of the eight trauma experiences, 540 (69.7%) had experienced between one and four of the events, and 171 (22.1%) had experienced five or more of the eight events. The incidence of each trauma type across groups is shown in Table 3.

**Table 3:** Incidence of trauma by level of victimization

	Zero (n = 64)	One to four (*n* = 540)	Five or more (*n* = 171)
Bullying	–	31.5%	77.2%
Neglect	–	54.3%	92.4%
Sexual assault	–	9.6%	42.1%
Physical assault	–	47.0%	93.6%
Threats of violence	–	60.4%	91.2%
Accidents	–	27.2%	56.1%
Disease	–	26.5%	67.8%
Death of family member	–	10.6%	27.5%

### Cannabis use during treatment

**Fig. 1:**
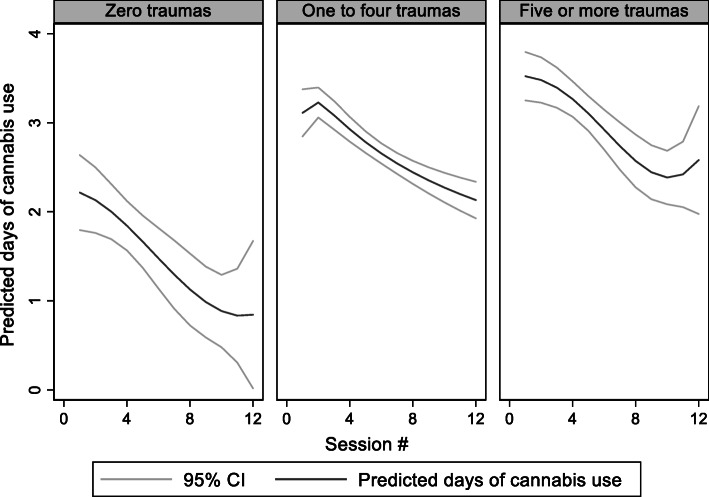
Days of cannabis use as a function of session by degree of poly-traumatization

Figure 1 shows days of cannabis use in the last week as a function of time.

The mean number of days declined from 4.04 on average at baseline (SD = 2.77) to 2.16 days at the twelfth session for the participants who completed all sessions (*n* = 134, SD = 2.88). The results of the Mixed Effects Tobit regression are shown in Table 4. The model Wald *χ*^2^ test was significant (*χ*^2^(13) = 268.1, *p* < 0.001).

**Table 4:** Predictors of days of cannabis use (Tobit regression, *n* = 775)

	Coefficient	*P*-value	95% CI	Coefficient	P-value	95% CI
Trauma						
Zero trauma types	Reference	–	–	Reference	–	–
1–4 trauma types	1.85	0.029	0.19; 3.51	1.56	0.064	-0.09; 3.22
5–8 trauma types	2.71	0.004	0.87; 3.54	2.06	0.030	0.20; 3.91
Session	−0.58	0.000	−0.72; −0.44	− 0.58	0.000	−0.71;-0.44
Trauma*session						
1–4 trauma types	0.29	0.010	0.05; 0.33	0.19	0.010	0.04; 0.33
5–8 trauma types		0.001	0.10; 0.40	0.25	0.002	0.09; 0.40
Female gender (reference: male gender)	–	–	–	1.13	0.033	0.09; 2.17
Age				0.12	0.158	−0.05; 0.29
Not in education, employment or training (reference: working or studying)				1.32	0.005	0.40; 2.24
Immigrant (reference: Danish)				0.17	0.810	−1.25; 1.60
Constant				2.05	0.441	−3.16; 7.26
Intraclass correlation:						
Site				< 0.01	–	–
Client				0.72	–	0.69; 0.75

In the unadjusted analyses, few or multiple trauma experiences were associated with a higher degree of cannabis use compared with no trauma history (one to four versus zero: *p* = 0.045; five or more versus zero: 0.010). With each session, days of cannabis declined (*p* < 0.001). The decline was less for those with a trauma history (one to four versus zero: *p* = 0.033; five or more versus zero: *p* = 0.004).

After controlling for confounders, clients who reported one to four types of trauma no longer differed significantly at baseline (coefficient = 1.65, CI − 0.09–3.22, *p* = 0.064), but clients who reported five or more types of trauma continued to show significantly more cannabis use at baseline (coefficient = 2.06, CI 0.20–3.91, *p* = 0.030). In terms of reductions in cannabis use over time, both clients who reported one to four of the trauma (coefficient = 0.19, CI = 0.04–0.33, *p* = 0.010), and clients who reported five or more of the trauma types (coefficient = 0.25, CI = 0.09–0.40, *p* = 0.002), showed a slower rate of decline in days of cannabis use compared to clients who did not report any of the trauma types, as indicated by significant group by time interactions.

The sensitivity analyses were not meaningfully different from the original analysis. As in the original analysis, both groups that reported trauma were less likely to reduce their cannabis use during treatment compared with those who did not report any of the trauma types.

### Session attendance

Clients with zero trauma types attended on average 6.5 sessions (SD = 4.7), clients with one to four trauma types attended on average 6.5 sessions (SD = 4.3), and clients with five or more trauma types attended on average 7.0 sessions (4.3). Table 5 shows the results of random effects logistic regression to predict session attendance.

**Table 5:** Predictors of session attendance (*n* = 775)

	Odds ratio	95% CI	P-value	Adjusted Odds Ratio	95% CI	P-value
Trauma	Reference	–	–	Reference	–	–
Zero trauma types	0.38	0.12; 1.17	0.092	0.36	0.12–1.07	0.067
1–4 trauma types	0.56	0.16; 1.91	0.353	0.54	0.16–1.83	0.320
5–8 trauma types	0.53	0.48; 0.58	0.000	0.52	0.47–0.58	0.000
Trauma*session						
1–4 trauma types	1.11	1.00; 1.23	0.050	1.12	1.01–1.24	0.036
5–8 trauma types	1.12	1.00; 1.25	0.055	1.13	1.01–1.26	0.038
Female gender (reference: male gender)	–	–	–	1.82	1.03–3.19	0.038
Age				1.18	1.07–1.29	0.001
Not in education, employment or training (reference: working or studying)				0.31	0.19–0.50	0.954
Immigrant (reference: Danish)				0.98	0.45–2.11	0.181
Constant				4.85	0.48–49.09	
Intraclass correlation:						
Site				0.04	0.01–0.11	
Client	0.87					
Var (cons)				9.80		

Compared with clients with zero trauma types, those with one to four (*p* = 0.036), and those with five to eight types (*p* = 0.038) were more likely to attend treatment at later sessions as indicated by significant session by group interactions.

**Fig. 2:**
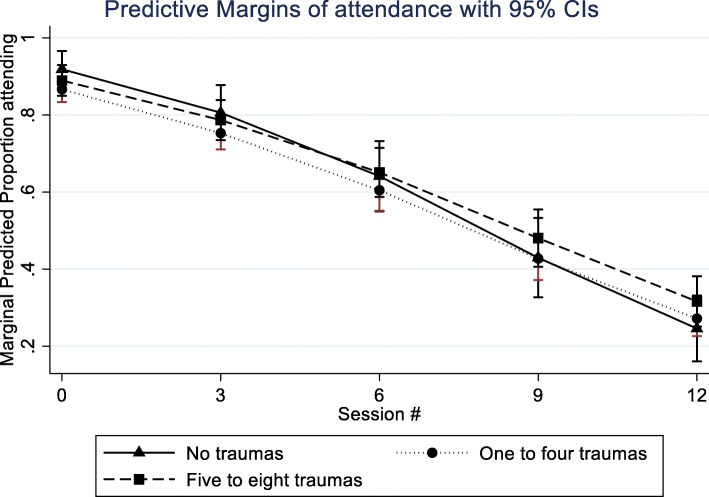
Marginal probability of attending sessions as a function of time and poly-traumatization

The marginal probability of attending sessions over time as a function of trauma victimization is shown in Fig. 2.

### Well-being

**Fig. 3:**
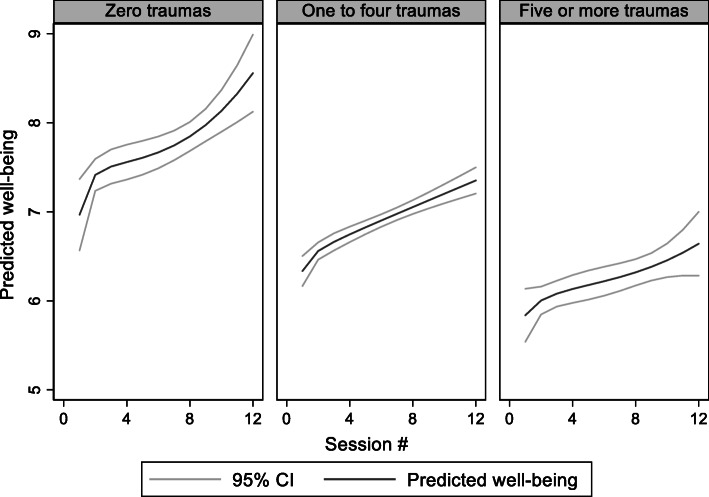
Self-reported well-being as a function of session by degree of poly-traumatization

A reliability analysis was carried out on the 3-item wellbeing scale. Cronbach’s alpha showed the scale to reach acceptable reliability, α = 0.71.The results of the logistic regression analysis for the association between well-being and the number of trauma types are shown in Table 6 and Fig. 3. In both the unadjusted (the second column of Table 6) and the adjusted models (column 4), trauma history was associated with lower overall well-being, but time by trauma interactions were non-significant, indicating that the rate of change did not differ between more or less trauma types. In all cases, well-being improved substantially over time in treatment.

**Table 6:** Predictors of self-reported wellbeing (*n* = 775)

	Coefficient	95% CI	P-value	Adjusted Coefficient	95% CI	P-value
Trauma						
Zero trauma types	Reference	–	–	Reference	–	–
1–4 trauma types	−0.73	−1.19–-.27	0.002	−0.62	−1.07– 0.17	0.007
5–8 trauma types	−1.20	−1.70–-0.70	0.000	−0.92	1.42–0.42	0.000
Session	0.09	0.05–0.13	0.000	0.09	0.05–0.13	0.000
Trauma*session						
1–4 trauma types	−0.00	−0.05–-0.04	0.891	−0.00	− 0.05–0.04	0.890
5–8 trauma types	−0.03	− 0.08–0.01	0.159	− 0.03	− 0.08–0.01	0.161
Female gender (reference: male gender)				−0.74	−1.00–-0.48	0.000
Age				−0.05	− 0.09–-0.01	0.022
Not in education, employment or training (reference: working or studying)				−0.21	−0.45–0.02	0.070
Immigrant (reference: Danish)				0.03	−0.32–0.28	0.860
Constant	7.08	6.65–7.51	0.000	8.96	7.94–9.97	0.000
Intraclass correlation:						
Site				< 0.01		
Client	0.53	0.49–0.56		0.51	0.48–0.55

### Sensitivity analyses

None of the sensitivity analyses yielded markedly different results.

## Discussion

On the basis of existing knowledge [59, 60], it is not surprising that the vast majority (91.8%) of the 775 participants in DUD treatment, had experienced one or more of the eight trauma types examined in this study, and that one out of five (22.1%) had experienced five to eight of the trauma types. However, considering the young age of the participants, the prevalence is indeed alarming and does strongly underline the importance of being very attentive to previous victimization in DUD treatment.

In this study, we found support for hypothesis-1 in that individuals who reported exposure to multiple types of trauma were less likely to reduce their use of cannabis in treatment and at a slower rate than individuals who reported fewer types or no types of trauma. In addition to this finding, our analyses also indicated that poly-traumatized youth enter DUD treatment with a higher use of cannabis than individuals with less types of trauma experiences.

Substance use severity at treatment entry has been negatively associated with treatment efficacy as indicated by higher substance use at follow-up [61], higher drop-out rate [62], relapse [63], and suicide attempt one year after treatment [64]. Similar to the present study, prior studies have also found a history of trauma victimization to be positively associated with severity of current substance use [65–67]. A higher consumption of cannabis at treatment entry, in itself, seems to provide a challenge for treatment of this specific group.

It is important to note that the associations between cannabis intake and number of trauma types were established without considering the psychological impact of the trauma experiences. If we had performed our analyses solely on a sample who reported being psychologically affected by their experiences, the association with cannabis may have been much stronger. A major hypothesis for the relationship between victimization and problematic substance use is that substance use is a coping strategy for dealing with the physical or emotional discomfort associated with the trauma of victimization. This includes using substances to avoid, escape or distract from the overwhelming distress associated with trauma-related memories [68, 69]. Although we did not measure traumatization or trauma symptoms in the present study, it is indeed possible that trauma symptoms are a causal factor behind the found association with a higher intake of cannabis and slower reduction of cannabis use in the poly-traumatized group. The probability of developing trauma related symptoms [4] and PTSD [12] increases with the number of victimization experiences and thus it is indeed likely that the rate of PTSD is higher in the poly-trauma group. In prior studies, PTSD symptoms have been shown to be associated with a higher cannabis intake and reduction of intake in treatment [65]. However, this association is very complex and the relationship between victimization and substance use has been shown, for instance, to vary across types of victimization experiences due to differences in emotional regulation [70]. Thus, the trajectories from victimization to substance use are many, and exposure to multiple traumatic events is one aspect in this complex interplay.

Due to the close relationship between trauma and substance-use, an increasing number of researchers and practitioners argue that the close relationship shared between trauma and substance misuse should be fully integrated into SUD treatment programs and that “Wherever possible, emphasis should be placed on the deep-rooted connection between trauma, mental health, and addiction” [71]. Although still not widely implemented, trauma focused interventions such as e.g. seeking safety [20, 25] has shown promising results, indicating that traumatized individuals in DUD treatment do benefit from a more integrative approach.

In order to target treatment at trauma-related issues, the first step is identification of such issues. Identifying individuals at particular risk in treatment is an important tool in treatment planning. It is possible that the level of traumatization may be a more precise risk indicator for treatment outcome than the number of trauma experiences in DUD treatment. However, we also need to relate to the current reality in DUD treatment settings. Although trauma symptoms and PTSD is far more prevalent in substance treatment settings than in the general population [72, 73] a very large proportion of clients with trauma symptoms remain unidentified and undiagnosed throughout treatment [72, 73]. In Denmark we do not routinely screen for PTSD in DUD treatment, and, to our knowledge, this is still not standard practice in most treatment centers around the world. It goes without saying that when clinicians in treatment facilities have no knowledge about clients´ trauma symptomatology, they cannot use it in treatment planning or risk assessments. However, in the majority of treatment facilities in Denmark we do screen for previous victimization experiences in DUD treatment. The present finding of the association between poly-traumatization and higher intake of cannabis at treatment entry and slower reduction throughout the treatment course may thus provide practitioners who holds knowledge about the victimization history of their clients but no knowledge on the degree of traumatization, indication of potential challenges in their treatment course.

In addition to specific factors associated with victimization, it is possible that slow progression in reduction of cannabis use in the poly-traumatized group could create a negative motivational spiral. It is well-known that the perception of making progress is important for personal motivation in any type of life change [74]. Clients and therapists often report lack of motivation as the main cause of non-successful treatment [75]. If poly-traumatized youth are experiencing less progress in terms of being able to reduce their intake of cannabis, their motivation for change in treatment may be affected negatively. Indeed, one study that examined motivational factors in treatment found that individuals who experienced slow or no progression towards their treatment goals more often had a perception of low competence which in turn led to less motivation for treatment [76]. In treatment, lack of motivation often leads to drop-out [77]. However, we did not find support for a higher drop-out rate among the poly-traumatized youth in the present study. It is, however, still possible that due to less progress in reduction of cannabis intake, motivational factors such as the perception of low competence may have negatively influenced this group’s ability to reduce their cannabis intake. Changes in motivation due to lack of progress could therefore be an important focus for traumatized patients in DUD treatment. Since, we did not measure any motivational factors, this hypothesis, however, remains unanswered in the present study.

In contrast to our hypothesis-3, number of sessions attended was positively associated with reported number of trauma types. In contrast, the groups that reported multiple trauma experiences (i.e. one to four and five to eight) were more likely to attend treatment sessions than the group of clients with no trauma experiences. This is indeed an encouraging finding, especially considering that several large-scale substance abuse treatment outcome studies have underscored the importance of length of time or retention in treatment as the most stable predictor of positive outcome [78, 79]. This finding is in fact consistent with the very modest associations between PTSD and retention in treatment for SUD presented in a recent narrative review. This review concluded that there is no clear pattern showing that patients with co-occurring PTSD are more likely to drop out from treatment than other patients with SUD [80]. However studies that have found associations between traumatization and retention in SUD treatment still suggest that there are important variables which modify the effect of trauma experiences on SUD treatment effect. In the above review, Hildebrand and colleagues suggest to further examine age, duration of treatment, types of substance use, length of follow-up, and method of assessment as potential explanatory factors for the differing results [80]. Also, we believe that more research into differences between specific victimization experiences, the victims´ perception of the experience and trauma symptoms would offer interesting insight in this context. For instance, it is likely that a large proportion of participants did not perceive their experiences as traumatic or have experienced persistent trauma symptoms [81]. A mixed method approach that also includes qualitative in-depth explorations of the reported trauma experiences, experienced trauma symptoms and experienced treatment outcomes would offer more detailed insight in differences between participants reporting many trauma experiences *and* trauma symptoms with the group reporting many trauma experiences and no or few trauma symptoms in relation to treatment attendance.

Also, our hypothesis-2 that the groups with a trauma history would experience less progress in wellbeing in DUD treatment compared to the group with no trauma history, was not supported either. The reported level of wellbeing at treatment entry (baseline) was, however, significantly lower in both the group with one to four types of trauma experiences as well as the group with five to eight types of trauma experiences, with the lowest score in the latter group. This finding indicates, that although the two groups had similar positive progression in symptoms throughout treatment, overall they had a lower well-being score, which in turn, combined with their higher intake of cannabis, suggests that they were more vulnerable throughout the treatment, as well as post treatment, compared to the group that reported no trauma experiences.

### Limitations

Firstly, self-reports of victimization experiences and substance use have their limitations. Specifically, questions on victimization that are not behavior-specific have been criticized because they may underestimate incidents since these questions assume a respondent will categorize their experience as abuse or be willing to self-identify as a victim [82]. It is argued, that people who have experienced physical or sexual abuse may not recognize that what they experienced was against the law or may be reluctant to categorize what they experienced as abuse [83]. However, this argument may be more relevant in surveys that examines the general population than in DUD treatment. Individuals in DUD treatment may be more prone to report victimization experiences, firstly because they are already in a developmental process in which they have recognized that they have a serious problem, and secondly, because that information could help their counsellor plan their treatment course. Due to the very high prevalence of victimization experiences in the present study, underreporting of victimization experiences do not seem to be an issue, but indeed more detailed and behavior-specific measures are recommended. Further, the validity of reported drug use has been questioned in multiple studies [84] and the measure we used for substance use has not been validated in a Danish context. It is indeed possible that both recall bias and underreporting may have influenced thhe drug use reports. However, we only asked for drug use within the past 7 days, which has minimized the influence of a recall bias. Even so, further research into the validity of the particular measure used in the present study is warranted. It is furthermore a limitation that we only measure the treatment effect on drug use via cannabis use. However, due to the low frequency of use of other drugs, it was not statistically meaningful to include these in this study. It is indeed possible that the interaction between traumatic experiences and treatment effect varies considerably depending on drug type or the presence of multiple drug use versus single drug use. More research into these variations is recommended. Also, we did not have data to examine the severity of trauma experiences and to which degree the participants were affected by their experiences. Consequently, we do not know if the level of traumatization is a causal factor and perhaps even the most important factor in the relationship between the number of trauma experiences and treatment outcome. Finally, an important limitation is that the findings do not generalize to participants who dropped out of treatment or cancelled multiple sessions. Thus, the results should be interpreted as representing the influence of trauma on progress during treatment, not beyond it.

## Conclusion

Overall, the results in this study support the hypothesis that poly-traumatized youth in DUD treatment may be harder to treat, due to a higher intake at treatment entry and a slower decline throughout treatment. In contrast to our hypotheses, analyses showed that the number of sessions attended and the progression in wellbeing in treatment were not affected by the number of trauma experiences. Since attendance has been found to be one of the most stable indicators for DUD treatment success, the non-significant association between trauma history and attendance is particularly an encouraging finding. However, specific psychological symptoms, social circumstances, and negative behaviors related to trauma, still emphasize the need for a specialized approach for victimized clients in DUD treatment.

## Data Availability

The datasets used and/or analyzed during the current study are available from the corresponding author on reasonable request.

## Abbreviations

PTSD: Post traumatic stress disorder
SUD: Substance use disorder
DUD: Drug use disorder

## Footnotes

1 Trial registration: ISRCTN27473213, 10.1186/ISRCTN27473213
